# Fibre-reinforced Cad/CAM post and cores: The new “gold standard” for anterior teeth with extensive coronal destruction?–A fully digital chairside workflow

**DOI:** 10.1016/j.heliyon.2023.e19048

**Published:** 2023-08-09

**Authors:** Jonas Adrian Helmut Vogler, Louise Billen, Kay-Arne Walther, Bernd Wöstmann

**Affiliations:** Justus Liebig University, Dental Clinic - Department of Prosthodontics, Schlangenzahl 14, 35392, Giessen, Germany

**Keywords:** CAD/CAM, Post and core, Intraoral scanner, Fibre-reinforced composite, Chewing simulation

## Abstract

**Objectives:**

Since one-third of persons suffer a dental trauma, treatment of anterior teeth using post and core (PC) is becoming important. In teeth with extensive destruction, cast PC (CPC) remain the “gold standard”, even though they lead to aesthetic impairment and have a mismatching elastic modulus to that of dentin. Prefabricated fibre-reinforced posts have elastic modulus similar to that of dentin but the accuracy of fit and mechanical stability are worse. This study was aimed to evaluate the deviation and mechanical performance of fibre-reinforced CAD/CAM PC (FRPC) fabricated in a fully digital chairside workflow, compared to those of CPC.

**Methods:**

On 30 teeth, a PC preparation was conducted, and a conventional and digital post impression were taken with an intraoral scanner. Fifteen teeth each were treated with CPC and FRPC, respectively. The deviation was evaluated by superimposing the datasets of the digitalised stone models and digital post impressions. Decementation and root fracture during chewing simulation were analysed by microscopy and X-ray. Statistical analysis was performed by pairwise comparison and Kaplan-Meier analysis.

**Results:**

The median deviation for the “coronal”, “middle” and “apical” were 14.5, 18.0 and 113.7 μm, respectively. The pairwise comparison for “coronal”/“middle” showed no significance (p = 0.465), whereas that for “coronal”/“apical” and “middle”/“apical” showed highly significant differences (p < 0.001). After chewing simulation, five decementations and two root fractures were detected for CPC. For FRPC, neither decementation nor root fracture were documented.

**Significance:**

Within the limitations of this study, FRPC performed significantly better than CPC.

## 1 Introduction

Even though the development of the adhesive technique in dentistry means that post and core (PC) is no longer necessary for all the teeth with loss of coronal tooth structure, it is still indispensable for cases with extensive defects [1,2]. Statistically, every third person suffers a dental trauma to the permanent dentition, which in many cases is related to severe damage to the tooth [3]. Thus, the restoration of the upper anterior teeth with PC is becoming important [[4], [5], [6]]. In addition to mechanical stability, aesthetics also play a decisive role with those cases; hence, many patients prefer the use of tooth-coloured and translucent materials in the anterior teeth [[7], [8], [9]]. Customised cast PC (CPC) has a high mechanical stability and is therefore indicated for extensive defects, because of their good accuracy of fit and monobloc structure that consists of a core and post components [[10], [11], [12], [13]]. However, due to the lack of translucency, CPC can lead to darkening of the tooth and its surrounding gingiva [9]. Moreover, the failure rate of PC in the upper anterior teeth is significantly higher than that in posterior teeth because of the non-axial masticatory load [14,15]. Therefore, many authors prefer the use of materials with an elastic modulus similar to that of dentin [7,8], to prevent root fracture [[16], [17], [18], [19]]. Other than CPC, prefabricated fibre-reinforced posts (PFRP) have a modulus of elasticity similar to that of root dentin and can positively influence the aesthetic appearance due to their translucency and colour [8,9,20]. Nevertheless, PFRP have a worse accuracy of fit compared to customised PC, and are less mechanically stable because of the interface between the PC parts [7,16,21,22]. Therefore, customised CPC remains the “gold standard” for treating cases of extensive loss of tooth structure [13,16].

Compared to other PC materials, such as metal or zirconia, the mechanical properties of fibre-reinforced composite differ depending on the direction of load to the fibre orientation [23]. PFRP have a unidirectional fibre orientation parallel to the posts long-axis, while recent fibre-reinforced CAD/CAM-materials have a multidirectional fibre orientation [23]. Suzaki et al. reported that the fracture resistance is 2.5 times higher when the direction of load is perpendicular to the orientation of the fibres, which is of high clinical relevance when the material is used for PC [24].

In the conventional workflow, a customized PC is waxed-up on a stone model based on the conventional impression of the prepared root canal and cast in alloy [25]. To use materials with an elastic modulus similar to that of root dentin for producing customized PC, it is necessary to scan the prepared root canal, because these materials are limited to a CAD/CAM workflow [16,26,27]. Recent developments of intraoral scanners (IOS) made it possible to mill customized PC in a fully digital workflow, by using the CAD/CAM-technology [16]. Leven et al. described that the accuracy of fit of CAD/CAM PC is within a clinically acceptable range [26]. Nevertheless, comparative studies between the “gold standard” of CPC and CAD/CAM PC made of a material with a similar elastic modulus to that of dentin, are lacking.

This in-vitro study was aimed to investigate the performance of customised CPC made of non-precious alloy fabricated in a conventional workflow, and fibre-reinforced CAD/CAM PC (FRPC) fabricated in a fully digital chairside workflow. The following null hypothesis were defined.

1. There is no significant difference between the datasets of the digitalised stone model and the digital post impression of the prepared root canal concerning the deviation in the coronal, middle and apical root canal areas.
2. There is no significant difference between the CPC and FRPC concerning root fracture and decementation during chewing simulation with thermocycling.

## 2 Materials & methods

In order to calculate the statistically significant sample size for the present study a power analysis with a pursued power of 95% was conducted. Due to the lack of similar comparative studies between different materials for PC concerning mechanical behaviour, the basis for this power analysis were the already published data for comparison between digital and conventional post impression [28,29]. The sample size was dependent on the number of measuring points in the root canal so that because of the high deviation in the reference studies in total 13 teeth with 9 measuring points would have allowed for a statistically significant analysis. Due to the scarce clinical data the sample size was increased to 15 teeth each for investigation group (C) and (D) in order to improve the statistical power.

**Fig. 1:**
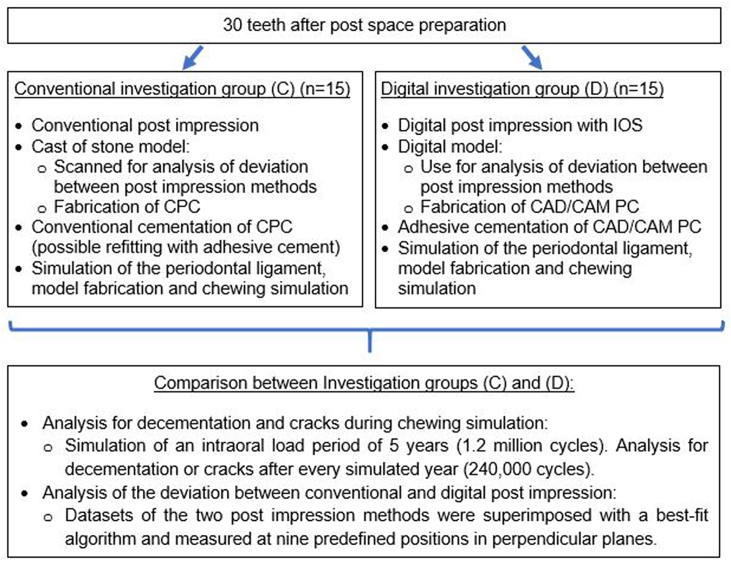
Schematic diagram of the experimental steps.

Fig. 1 gives an overview over the experimental steps of the present study which are described in detail in the following sections.

### 2.1 Tooth preparation

For this study, 30 single-rooted teeth with comparable sizes of 15 mm length and 6 mm width (±1.0 mm) were selected [30,31]. The teeth had been extracted for therapeutic reasons and their use for research purposes was approved by the ethics committee of the Medical Faculty of Justus Liebig University Giessen (Reg. No. 143/09) . The crowns were removed up to 2 mm above the cementoenamel junction using a disc grinder with water cooling. Then, a chemo-mechanical endodontic treatment was processed by using 3.0% sodium hypochlorite and a system of rotary files (F-360 ISO 15–45, Komet, Germany). This was followed by filling with gutta-percha (ISO 45, Taper 0.04, Coltène/Whaledent AG, Switzerland) and sealer (AH Plus, Dentsply DeTrey, Germany), using the one-point technique. After the cure time of the sealer (24 h), the post space preparation was conducted using the ER System (ISO 90, Komet, Germany) at a length of 10 mm. Additionally, a rotation lock by means of a 3 mm box at the root canal entrance and a chamfer line was prepared with diamond burs under constant water cooling. Subsequently, the roots were examined, and teeth with infractions, cracks and root caries were excluded. The analysis was carried out under a digital light microscope (Smartzoom 5, Zeiss, Germany) and based on X-ray findings.

### 2.2 Conventional/digital PC impression and fabrication

**Fig. 2:**
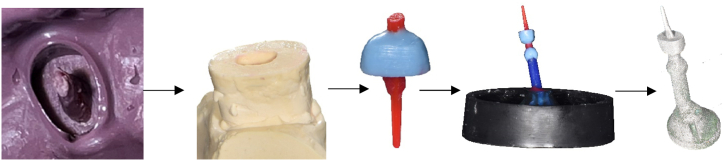
Conventional workflow for fabricating PC in group C. From left to right: conventional post impression, stone model, wax up of PC, PC modelling embedded, CPC.

On every teeth, one conventional and one digital post impression were taken as described below. Subsequently, the 30 teeth were divided into two investigation groups (conventional and digital): 1. Conventional (C): For 15 teeth, CPC, according to the conventional workflow [25] was fabricated as follows: the conventional post impression was taken using a polyether material (Impregum, 3 M GmbH, Germany) with a resin post (ER CAST-Stift, ISO 90, Komet, Germany). The impression was transferred to a stone model (Implantat-rock, Picodent, Germany) which was subsequently scanned with an IOS (Primescan, Version 5.2.3, Dentsply Sirona, Germany) for the analysis of deviation between the two post impression methods (see chapter 2.6) before using for PC modelling. The gypsum powder was mixed in a vacuum machine for 60 s with distilled water in the ratio that was recommended by the manufacturer. After setting time of the stone model for 24 h the PC modelling was performed by use of a new resin post and wax (Dentaurum, Germany). The core part in all PC was designed with a height of 5 mm. The wax up was embedded in an investment material (Heravest Onyx, Kulzer, Germany) for casting a non-precious alloy PC (Wirobond C, Bego, Germany) by use of the lost wax technique. Fig. 2 shows the conventional workflow for fabrication of CPC.

**Fig. 3:**
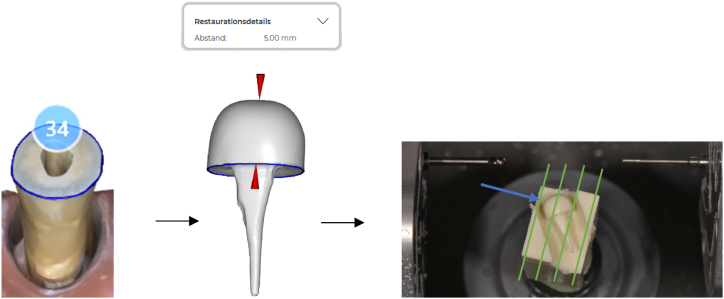
Digital workflow for FRPC fabrication in group D. From left to right: digital post impression, virtual PC design (red arrows: 5 mm height of the core part), FRPC in the milling unit: Orientation of glass fibre mats in the CAD/CAM block (green lines) perpendicular to the direction of load during chewing simulation (blue arrow).

2. Digital (D): For 15 teeth, FRPC, according to the fully digital chairside workflow was fabricated as follows: the digital PC impression was taken using an IOS. Subsequently, the STL-dataset was used for designing the PC at a core height of 5 mm, using the IOS software, to allow for comparison with CPC for group C during chewing simulation. The cement gap parameter was set to 50 μm in the software. The FRPC was fabricated in <10 min in a fully digital workflow using a milling unit that was developed for chairside restorations (MCXL, Dentsply Sirona, Germany) and a fibre-reinforced CAD/CAM composite with multidirectional orientation of glass fibre mats (Trinia, Bicon Europe Ltd., Germany). To ensure that the direction of load during chewing simulation is perpendicular to the glass fibre mats, the FRPC orientation was rotated by 45° in the CAD/CAM block. Fig. 3 shows the digital workflow for FRPC fabrication and the orientation of fibres (green lines) in relation to the direction of load (blue arrow).

Table 1 illustrates the materials with the elastic modulus used for CPC and FRPC fabrication in group (C) and (D), respectively.

**Table 1:** Materials for PC fabrication.

Investigation group	Material	Product name	Product name	Elastic modulus
Conventional (CPC)	non-precious alloy	Wirobond C	Bego	180 GPa
Digital (FRPC)	fibre-reinforced CAD-CAM composite	Trina	Bicon	18.8 GPa

Elastic modulus of dentin: 18.6 GPa [5].

### 2.3 Fitting of PC in the root canal

The PC for both investigation groups were blasted with aluminium oxide and then cleaned in an ultrasonic bath with ethanol (ethanol 70% (V/V) Hofmann's, Hofmann & Sommer GmbH und Co. KG, Germany). The prepared root canal was cleaned with a diamond-coated hand instrument (Aufrauinstrument 196D, ER System, Komet, Germany). The debris was removed by rinsing with 3% sodium hypochlorite solution. Subsequently, the root canal was dried with paper points.

CPC of group (C) were fitted with glass ionomer luting cement (Ketac™ Cem Aplicap™, 3 M GmbH, Germany) according to an established conventional workflow [25]. The cement was filled into the lumen with a probe and was applied to the post part of CPC. Under constant finger pressure, the CPC was pressed into the final position and held for 7 min of setting time. Subsequently, the excessive cement was removed.

The FRPC of group (D) were fitted with an adhesive cement (PANAVIA V5, Kuraray Noritake, Kuraray Noritake, Japan) according to the manufacturer's instruction [32]. The first primer (CLEARFILTM CERAMIC PRIMER PLUS, Kuraray Noritake, Japan) was applied on the post part of PC. The second primer (PANAVIA V5 Tooth Primer, Kuraray Noritake, Japan) was applied for 20 s on the luting surfaces of the tooth. The excessive primer in the canal was removed with paper points and then dried by a gentle air stream. The adhesive cement (PANAVIA V5 Paste, Kuraray Noritake, Japan) was applied to the post part of PC. Under constant finger pressure, the PC was pressed into the final position and held for 7 min of setting time. Excessive cement was removed with a foam pellet. In addition, a gel (OXYGUARD™, Kuraray Noritake, Japan) was applied circularly to the margins to prevent the formation of an oxygen inhibition layer. Subsequently, all surfaces were light-cured for 20 s, and the gel was removed.

After PC fitting, all 30 teeth were once again examined for infractions and cracks by digital light microscope analysis and X-ray. In addition, the transition area from PC to the tooth was documented by images taken using the digital light microscope software as a reference for the analysis of cracks throughout chewing simulation (T0).

In case of a decementation during chewing simulation, PC and the prepared root canal were cleaned of cement residues and examined for infractions and cracks by microscopic analysis and X-Ray. Teeth with infractions or cracks were excluded from further investigation. If no infraction or crack was detected, CPC of group (C) were adhesively refitted according to the luting protocol described for FRPC in group (D). This procedure is in line with established clinical practice [33].

### 2.4 Simulation of periodontal ligament, model fabrication and chewing simulation

**Fig. 4:**
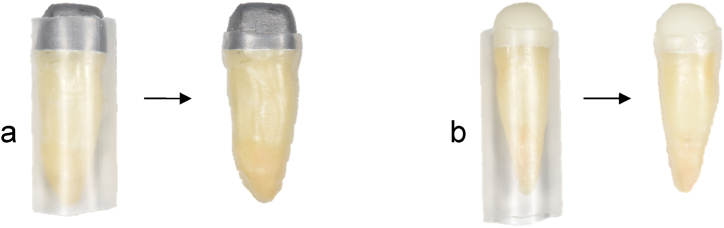
Simulation of periodontal ligament using a shrink tube (a: sample teeth for group C; b: sample teeth for group D).

To simulate the periodontal ligament for chewing simulation, the roots were fixed in a shrink tube (DERAY®-KY 175, SHAWCOR, Canada) that is comparable to the human periodontal ligament regarding thickness and elastic modulus [34,35]. For shrinking, the tube was heated evenly using a hot air dryer, so that the root was covered without any gaps. Subsequently, the tube was cut off at the cementoenamel junction (Fig. 4).

**Fig. 5:**
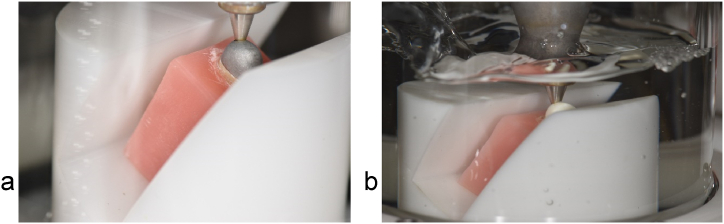
Chewing simulation (a: CPC of group C in the mount of the chewing simulator at an inclination of 45° to the axis of masticatory load; b: FRPC of group D during chewing simulation under thermocycling in a water bath).

For chewing simulation, all sample teeth were embedded in a block of acrylic resin fitted into the mount of the chewing simulator at an inclination of 45° to the axis of masticatory load. Therefore, the roots were polymerised in the block, up to 2 mm below the cementoenamel junction (Fig. 5). Subsequently, three measurements using *Periotest* (Periotest Classic type 3218, Medizintechnik Gulden e.K., Germany) were processed, to verify that the simulation of the periodontal ligament was equivalent to that of a clinical tooth mobility grade 0. If the mean value was outside the range of −08 to +09, the tooth was excluded from further investigation. Then, the chewing simulation was processed for 1.2 million cycles of load under thermocycling in a water bath (SD Mechatronik Chewing Simulator CS-4.8; SD Mechatronik GmbH, Germany) (Fig. 5), which equals an intraoral load period of 5 years [36].

Table 2 illustrates the set parameters of the chewing simulator.

**Table 2:** Set parameters of the chewing simulator.

Loading force [N]	50
Force frequency [Hz]	1.6
Chewing speed [mm/s]	60
Mouth opening distance [mm]	2.0
Thermocycling temperature	5 °C/55 °C, 95 s each
Antagonist	Stainless steel cone/point radius: 30°/Vickers hardness: 385 R1, SD Mechatronik GmbH, Germany

### 2.5 Analysis for decementation and cracks during chewing simulation

After every 240,000 cycles of load, simulating 1 year of intraoral loading period (T1-T5), the chewing simulation was stopped and the transition area of the sample teeth was analysed for infractions or cracks using a digital light microscope and X-Ray. In case of an infraction or crack, the sample was classified as “root fracture”, the simulated survival time (T1-T5) was documented and the sample was excluded from further investigation. In cases of decementation the survival time (T1-T5) was documented and PC was recemented as described in paragraph 2.3 in order to follow and examine the clinical established practice after loss of retention of PC. After recementation, the chewing simulation was restarted from the beginning for 1.2 million cycles of load. The decementation, infractions or cracks analysis were repeated after every simulated year of intraoral loading period (240,000 cycles of load).

### 2.6 Analysis of deviation between conventional and digital post impression

**Fig. 6:**
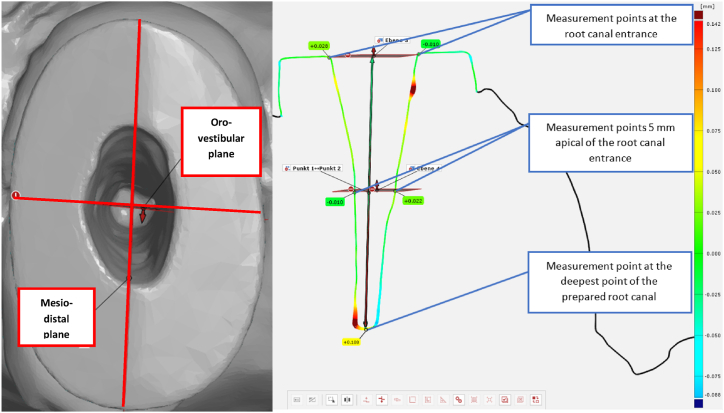
Left: Mesio-distal (largest diameter of the tooth circumference) and oro-vestibular (smallest diameter of the tooth circumference perpendicular to plane in mesio-distal direction) plane construction. Right: Measuring points of deviation between the da**tasets of the conventional and digital post impression on the oro-vestibular plane.**

For the analysis of deviation between the conventional and digital post impression, the unchanged stone models before PC modelling were scanned using the same IOS that was used for digital post impressions. Subsequently, the corresponding datasets (digitalised stone model and digital post impression of the prepared root canal) were superimposed using a 3D analysis software (Version V8 SR1 2020, GOM inspect, GOM GmbH, Germany) and a best-fit algorithm. The deviation of the datasets was measured at nine predefined points of the root canal using the “measuring flag” function of the analysis software. Therefore, two perpendicular planes were constructed along the lumen of the prepared root canal with the software tool “plane in viewing direction”. One plane each was set in mesio-distal (largest diameter of the tooth circumference) and oro-vestibular direction (smallest diameter of the tooth circumference perpendicular to plane in mesio-distal direction), respectively. For each plane, four measurements were taken as follows: two each at the points of intersection with the root canal entrance and with the root canal wall 5 mm apical of the root canal entrance, respectively. Measurement point number nine was the deviation between the datasets at the deepest point of the prepared root canal. Fig. 6 shows an example of the plane construction and measurement of deviation between the two datasets.

### 2.7 Statistical analysis

IBM SPSS Statistics for Windows, version 26 (IBM Corp., Armonk, NY, USA) was used for statistical analysis. Decementations and root fractures during chewing simulation over a 5-year simulated time of intraoral loading, were assessed using Kaplan-Meier analysis. Cases in which neither decementations nor root fractures occurred were rated as “censored cases”. The pairwise comparison between the two investigation groups was assessed using the log-rank test. For statistical analysis of the deviation between the datasets of the conventional and digital post impression, the measuring points at the entrance, 5 mm apical and deepest point of the root canal, were assigned to the “coronal”, “middle” and “apical” category, respectively. A pairwise comparison (median test) was performed to investigate significant differences between the three measurement categories. Due to alpha error accumulation, p-values were corrected using the Bonferroni method and level of significance was set at p < 0.05.

## 3 Results

### 3.1 Deviation between conventional and digital post impression datasets

The calculated medians of the measurement categories were 14.5, 18.0 and 113.7 μm for “coronal”, “middle” and “apical”, respectively. The results of the pairwise comparison between “coronal” and “middle” showed no significant difference. However, a highly significant influence was shown for the pairwise comparisons between “coronal” and “apical” as well as “middle” and “apical”. The p-values of the pairwise comparisons between the measurement categories and median values with the corresponding standard deviations are illustrated in Table 3.

**Table 3:** Significant influences of the pairwise comparison (median test).

Deviation between the conventional and digital post impression		
**Categories**	**Median** ± **standard deviation [μm]**	**P-value of median test**
Coronal (CO)	14.5 ± 24.8	0.465 (MI) < 0.001 (AP)^a^
Middle (MI)	18.0 ± 21.7	0.465 (MI) < 0.001 (AP)^a^
Apical (AP)	13.7 ± 159.8	<0.001 (CO)^a^ <0.001 (AP)^a^

^a^ =Significant influence.

**Fig. 7:**
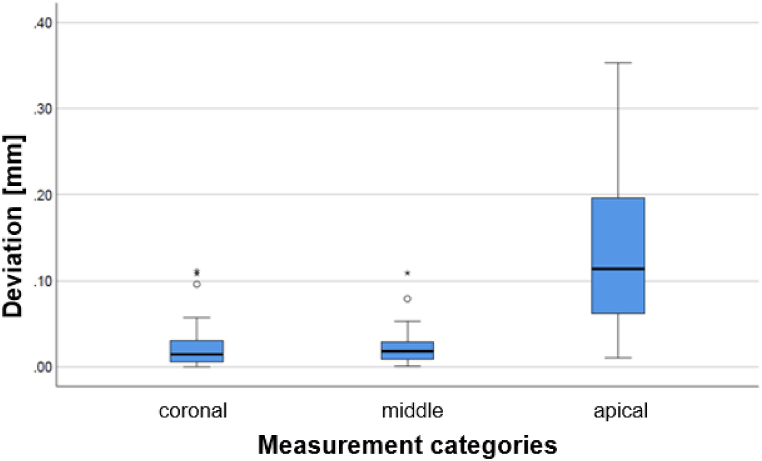
Box-plot diagram of deviation between the conventional and digital post impression datasets distributed according to the three measurement categories (“coronal”, “middle” and “apical”).

Moreover, the deviation increased from “coronal” to “apical” with the highest divergence in “apical” category. Fig. 7 is the box-plot diagram of the deviation between the conventional and digital post impression datasets distributed according to the “coronal”, “middle” and “apical” measurement categories.

Hence, the first null hypothesis of no significant difference between the digitalised stone model and digital post impression datasets of the prepared root canal regarding the deviation among the coronal, middle and apical root canal areas was partially rejected.

### 3.2 Decementations and cracks after chewing simulation

**Fig. 8:**
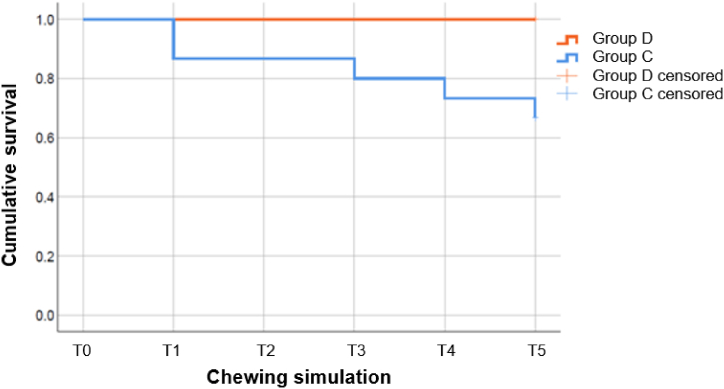
Kaplan-Meier curve of the chewing simulation: FRPC of group D (orange) and CPC of group C (blue).

In group (D) (FRPC fabricated in a fully digital chairside workflow), neither decementations nor root fractures were detected during the 5-year chewing simulation of 1.2 million cycles. Therefore, the simulated 5-year survival rate in group D was 100%. In group (C) (CPC fabricated in a conventional workflow), five CPC lost retention during chewing simulation. Three decementations occurred after 240,000 (T1), one after 720,000 (T3) and one after 960,000 (T4) cycles of load. Moreover, two PC in group C showed dentinal cracks after chewing simulation (one each at T1 and T5). Therefore, the simulated 5-year survival rate in group C was 65.0% with a mean survival time of 4.05 simulated years of intraoral loading period (standard deviation = 1.64). For PC after adhesive recementation (five CPC in group C), neither decementations nor dentinal cracks were recorded. Fig. 8 illustrates the Kaplan-Meier survival curve for PC after chewing simulation. The pairwise comparison (log-rank test) showed a significant difference (p = 0.042) between groups (C) and (D).

Therefore, the second null hypothesis of no significant difference between CPC and FRPC concerning root fracture and decementation after chewing simulation with thermocycling was rejected.

## 4 Discussion

The deviation between the two post impression methods and the mechanical behaviour during chewing simulation in a set up that is comparable to that of the clinical treatment of teeth with extensive coronal destruction, was investigated. Especially for anterior teeth, FRPC have the potential to improve dental treatment regarding the aesthetics and mechanical stability because of the translucency and similar elastic modulus to those of dentin. To the best of the authors knowledge, there has been no comparable study in dental literature investigating CAD/CAM PC that combines the advantages of PFRP, that are widely used in dental practice, with those of customised PC, in a fully digital chairside workflow.

### 4.1 Method

Even though the elastic modulus of alloy used for fabrication of CPC is significantly higher than that of dentin [37,38], customised CPC is still regarded as the “gold standard” for treating teeth with severe coronal destruction because of its excellent accuracy of fit [13,16]. PFRP have a mechanical behaviour similar to that of dentin but are less stable due to the interface between the PC parts and the poor accuracy of fit in the prepared root canal [7,16,21,22,38]. Therefore, to combine the advantages of both types of PC, the accuracy of fit has to be a major assessment criterion when it comes to evaluation of further developed PC treatment options. In the present study, the deviation between the two investigated workflows was measured by superimposing the two datasets with a best fit algorithm, which is an established procedure in dental literature [26,28,[39], [40], [41]]. Therefore, the software superimposes the datasets automatically to minimise the discrepancy between the point clouds [42]. *O'Toole* et al. investigated the accuracy of superimposing two datasets using the best fit algorithm, a three-point superimposition and the same coordinate system, for both scans [43]. Although the superimposition of the same coordinate system for both scans resulted in the smallest error, this procedure was described as non-clinically feasible due to the lack of reference structures for the coordinate system. The use of the best fit algorithm led to less discrepancies compared to the three-point superimposition [43]. *Revilla-León* et al. confirmed this conclusion and found the highest accuracy and precision for the best fit algorithm in a clinically feasible set up [42]. Most studies dealing with the evaluation of the accuracy of fit of fixed dental prothesis used the analogue replica technique [44,45] or microcomputed tomography [46] to measure the cement gap. Nevertheless, these techniques are not feasible for PC because of the fragile impression of the cement gap in the prepared root canal and the artefacts caused by X-Ray with alloy restorations [46]. Thus, for evaluating the deviation between the two post impression methods in the present study, the superimposition of the datasets with the best fit algorithm was the most suitable method. To minimise the influence of the scanner system, the stone models were digitalised using the same IOS that was used for the digital post impressions. *Chen* et al. showed for the IOS used in the present study, the highest precision in comparison to that of another IOS and an extraoral laboratory scanner system [47]. *Ender* et al. confirmed these findings in a comparative study between seven different IOS systems [48]. *Primescan* has a higher depth of focus compared to that of other IOS. Therefore particularly suitable for digital post impression in a fully digital chairside workflow for CAD/CAM PC, especially because it is part of an established system that was developed for fabrication of chairside fixed dental protheses [28].

In addition to high accuracy of fit in the prepared root canal, the mechanical behaviour of PC is also non-negligible because the teeth and restorations are exposed to continuous stresses from chewing movements and parafunctions in clinical use [49]. The artificial oral environment and changing temperatures within the oral cavity affect the retention of PC as well as the PC material itself [32,50]. Therefore, preclinical survival simulation in a set up that is comparable to the clinical situation is essential in terms of material fatigue and further development of new treatment options [49]. Thus, many authors in dental literature reported using chewing simulators to analyse the mechanical behaviour of new materials or workflows, similar to those performed in the present study [49,51]. *Rosentritt* et al. described that a simulation of the periodontal ligament is necessary during chewing simulation, since the omission of this resulted in a three times lower susceptibility to fracture, which decreases the comparability to the clinical situation [52]. For this, in many dental literature, a thin layer of impression material with a comparable thickness but different mechanical properties than those of the human periodontal ligament was used [36,52,53]. Moreover these previous studies did not examine the simulation of the periodontal ligament for mobility grade before chewing simulation. In the present study, the artificial periodontal ligament was made of a shrink tube with a thickness and mechanical properties similar to those of the intraoral situation in anterior teeth [34,35]. Moreover, a check using *Periotest* was performed before chewing simulation. Nevertheless, it has to be mentioned that the simulation of the periodontal ligament is still a limitation and a possible influencing factor on the results of the present study. Since the periodontal ligament shows a complex elastic behaviour it is obvious that the simulation with a polymeric shrink tube can only be an approach to the clinical situation but to the best knowledge of the authors there is no better simulation of an artificial periodontal ligament described in dental literature. Another limitation of the present study was that the luting protocol was not the same for both investigation groups at the beginning of the chewing simulation. The reason for this was that the study aimed to compare the conventional workflow for CPC, which includes conventional fitting with glass ionomer cement [25], to the new fully digital chairside workflow for CAD/CAM PC made of *Trinia*. According to the manufacturer's instruction fitting of *Trinia* is limited to adhesive cementation. That is why the luting protocol was not the same at the beginning of chewing simulation. After possible decementation also CPC were refitted adhesively following the protocol of investigation group (D) which is in line with established clinical procedure in our clinic [33]. In order to minimise the influence of chewing simulation before decementation on the mechanical behaviour after refitting of PC the teeth were critically examined for infractions and cracks and excluded if damage was detected. Nevertheless, it has to be mentioned that an influence of chewing simulation before decementation cannot be completely excluded and is therefore an inevitable limitation of the present study.

Many studies investigated the mechanical behaviour of PC using a covering crown since the ferrule effect is a known significant factor for clinical success [2,49]. In the present study, a ferrule design was deliberately omitted to investigate the uninfluenced effect of the material on loss of retention and root fracture. This approach is described by other studies as well [54,55]. In the present study, the evaluation of decementation and root fracture after chewing simulation was analysed using a digital light microscope and X-Ray. *Iemsaengchairat and Aksornmuang* investigated CAD/CAM PC and conventionally CPC and also used microscopic analysis and X-Ray for evaluation, since this approach makes it possible to detect microcracks in root dentin, which can hardly be seen by visual inspection [31].

### 4.2 Results

In the present study, the median deviation between the two post-impression methods in the “apical” category was 113.7 μm. *Pinto* et al. investigated the accuracy of fit of post impressions using preparation lengths of 8.5 mm–9.8 mm [29]. They described that digital post impression using an IOS led to a 1.83 mm shorter impression depth than a conventional post impression. However, this previous study used an outdated IOS with a smaller depth of focus. *Leven* et al. found a tenfold smaller deviation using the latest generation of a modern IOS [26]. Nevertheless, *Elter* et al. described an apical deviation of 357.1 μm for digital post impression using the *Primescan* compared to a conventional post impression [28]. One reason for the higher deviation compared to that of the present study could be the software version. *Elter* et al. used *Primescan* version 5.0.0, whereas, in the present study, version 5.2.3 was used. The influence of the IOS software on the precision of digital impressions has been described in dental literature [56]. This indicates that for fabrication of CAD/CAM PC in a fully digital workflow, the IOS needs to have a hardware with a high depth of focus and an updated software version to facilitate good accuracy of fit. In the present study the deviation increased from “coronal” to “apical” within a clinically acceptable range [26]. This result is in line with other authors in dental literature although in the present study, the deviation is to a much smaller extent [26,28]. Another reason for the high divergence in “apical” measurement category values could be because of the vulnerability of the conventional post impression. Since with an analogue impression, the complete representation until the deepest point of the prepared root canal can hardly be checked, whether an impression material did not reach every part of the preparation resulting in a CPC with a large cement gap in the apical area, cannot be excluded. The authors recognised this in some teeth with CPC on the proceeded X-ray. In those cases, the digital post impression might have led to a better accuracy of fit in the apical area; however, since the conventional post impression dataset was set as the “reference” and the pairwise comparison test in the present study was limited to the absolute values, this could not have been taken into the account. The described procedure might be a weakness of the present study and should be further investigated in future studies; however, it was inevitable in the context of investigating the significant differences between the two workflows. Concerning the analysis of decementation and root fracture during chewing simulation with thermocycling in the present study, five CPC lost retention, and two showed dentin cracks in the transition area between PC and root. For FRPC, neither decementation nor root fracture was detected. *Hayashi* et al. investigated the in-vitro fracture resistance of pulp-less teeth restored using PFRP and metallic posts. The inclination of the axis of masticatory load to the axis of the post was 45°, similar to that of the present study. The authors found a significantly higher fatigue limit for teeth restored using fibre-reinforced posts and presumed a similarity of elastic modulus between post and dentin for this result [19]. *Ferrari* et al. compared the clinical survival of CPC and PFRP retrospectively over an observation period of 4 years. CPC showed root fractures in 9% of cases, whereas no fracture occurred in the group of teeth restored using fibre-reinforced posts [57]. Contrary to this, *Altitinchi* et al. found a 100% failure rate because of fractures after chewing simulation with thermocycling for FRPC made of *Trinia*. However, the previous study did not consider the orientation of the fibre mats to the axis of the load and did not rotate PC in the CAD/CAM block, different from that of the present study [49]. *Suzaki* et al. described the significant influence of fibre orientation for fracture toughness especially when the material was used for PC [24]. This could be the reason for the different results of FRPC made of *Trinia* in the present study compared to that by Altinchi et al. Most studies dealing with the survival probability of PC found the loss of retention as the most common cause of failure [7,25,58], which is in line with the results of the present study. Even though the loss of retention is described as a relative cause of failure because PC can often be recemented without additional effort [25], it can still increase the risk for tooth loss. Ona et al. described that the mismatch of the elastic modulus appears to be a factor responsible for decementation of metallic PC from the root canals, with a potential increase in the risk of root fractures. This is in line with the findings of the present study because root fracture was only documented for CPC cemented with glass ionomer cement. After recementation of CPC with an adhesive cement, no root fracture or further decementation was detected. This indicated that adhesive cementation of CPC seems to have an advantage over conventional cementation with regard to the retention in the root canal as well as decreasing risk of root fracture.

## 5 Conclusion

The fully digital chairside workflow for CAD/CAM PC described in the present study has the potential to combine the advantages of a chairside treatment with PFRP and customised CPC. Within the limitations of this study, the fibre-reinforced CAD/CAM composite (*Trinia*) can be used to fabricate customised PC for anterior teeth with extensive coronal destruction in a fully digital chairside workflow with superior mechanical behaviour and accuracy of fit compared to the “gold standard” of CPC. Moreover, the results showed that even CPC should be cemented adhesively, to decrease the risk for loss of retention and root fracture.

## Funding

The work was supported by the Department of Prosthodontics, Justus Liebig University in Giessen, Germany and the German Society of Prosthodontics.

## Ethical approval

Use of extracted teeth for research purposes (Reg. No. 143/09).

## Declaration of competing interest

The authors declare that they have no known competing financial interests or personal relationships that could have appeared to influence the work reported in this paper.
